# MTF1 Is Essential for the Expression of MT1B, MT1F, MT1G, and MT1H Induced by PHMG, but Not CMIT, in the Human Pulmonary Alveolar Epithelial Cells

**DOI:** 10.3390/toxics9090203

**Published:** 2021-08-29

**Authors:** Sang-Hoon Jeong, Cherry Kim, Jaeyoung Kim, Yoon-Jeong Nam, Hong Lee, Ariunaa Togloom, Ja-Young Kang, Jin-Young Choi, Hyejin Lee, Myeong-Ok Song, Eun-Kee Park, Yong-Wook Baek, Ju-Han Lee, Ki-Yeol Lee

**Affiliations:** 1 Medical Science Research Center, Ansan Hospital, Korea University College of Medicine, Ansan-si 15355, Korea; saibog21@korea.ac.kr (S.-H.J.); iioiilloll@korea.ac.kr (J.K.); nyj90504@korea.ac.kr (Y.-J.N.); hlee3383@korea.ac.kr (H.L.); aina_tog@yahoo.com (A.T.); streetlife12@naver.com (J.-Y.K.); qaz9201@naver.com (J.-Y.C.); hyejin2.bio@gmail.com (H.L.); choa45@nate.com (M.-O.S.); 2 Department of Radiology, Ansan Hospital, Korea University College of Medicine, Ansan-si 15355, Korea; cherrykim0505@gmail.com; 3 Department of Medical Humanities and Social Medicine, College of Medicine, Kosin University, Busan 49267, Korea; eunkee.park@gmail.com; 4 Humidifier Disinfectant Health Center, Environmental Health Research Department, National Institute of Environmental Research, Incheon 22689, Korea; sideworks@korea.kr; 5 Department of Pathology, Ansan Hospital, Korea University College of Medicine, Ansan-si 15355, Korea

**Keywords:** polyhexamethylene guanidine, chloromethylisothiazolinone, metallothionein 1, metal regulatory transcription factor 1, human pulmonary alveolar epithelial cells

## Abstract

The inhalation of humidifier disinfectants (HDs) is linked to HD-associated lung injury (HDLI). Polyhexamethylene guanidine (PHMG) is significantly involved in HDLI, but the correlation between chloromethylisothiazolinone (CMIT) and HDLI remains ambiguous. Additionally, the differences in the molecular responses to PHMG and CMIT are poorly understood. In this study, RNA sequencing (RNA-seq) data showed that the expression levels of metallothionein-1 (MT1) isoforms, including *MT1B*, *MT1E*, *MT1F*, *MT1G*, *MT1H*, *MT1M*, and *MT1X*, were increased in human pulmonary alveolar epithelial cells (HPAEpiCs) that were treated with PHMG but not in those treated with CMIT. Moreover, upregulation of MT1B, MT1F, MT1G, and MT1H was observed only in PHMG-treated HPAEpiCs. The protein expression level of metal regulatory transcription factor 1 (MTF1), which binds to the promoters of MT1 isoforms, was increased in PHMG-treated HPAEpiCs but not in CMIT-treated HPAEpiCs. However, the expression of early growth response 1 (EGR1) and nuclear receptor superfamily 3, group C, member 1 (NR3C1), other transcriptional regulators involved in MT1 isomers, were increased regardless of treatment with PHMG or CMIT. These results suggest that MTF1 is an essential transcription factor for the induction of MT1B, MT1F, MT1G, and MT1H by PHMG but not by CMIT.

## 1. Introduction

Fatal lung injury is one of the most harmful effects caused by the inhalation of humidifier disinfectants (HDs), which are compounds used to prevent the growth of microorganisms such as bacteria and fungi, inside the humidifiers. Polyhexamethylene guanidine (PHMG), oligo(2-(2-ethoxy)ethoxyethyl) guanidinium chloride, chloromethylisothiazolinone (CMIT), and methylisothiazolinone are the most common HDs [1,2]. Although exposure to PHMG has clearly resulted in lung injuries, such as interstitial pneumonitis and fibrosis, CMIT is not considered to be correlated with lung injuries in humans [1,2]. Although a few clinical and animal studies have reported that lung damage after inhalation of CMIT is lower than that observed after inhalation of PHMG [3,4], the differences in the molecular mechanisms between PHMG and CMIT have not yet been fully elucidated.

In vitro studies have revealed the significant involvement of PHMG in apoptotic cell death. The increased levels of reactive oxygen species after PHMG inhalation exert cytotoxic effects, such as membrane and DNA damage [5,6,7,8]. Furthermore, PHMG has been shown to cause endoplasmic reticulum stress via mitochondrial dysfunction [9]. Exposure to PHMG upregulates the expression of genes involved in various biological processes including apoptosis, autophagy, fibrosis, and cell cycle and downregulates the expression of antioxidant-related genes [10]. PHMG augments the epithelial-mesenchymal transition-related microRNA expression, which may play a role in lung fibrosis [11]. Apoptosis and inflammation have been reported after CMIT exposure; however, CMIT has a weaker association with cellular toxicity than PHMG [12]. Moreover, no study has compared the cytotoxic mechanisms of PHMG with those of CMIT.

Metallothioneins (MTs) are small cysteine-rich proteins that bind to intracellular metal ions, such as Zn^2+^, Cu^2+^, and Cd^2+^, to maintain cellular metal homeostasis. The genes encoding these proteins are expressed in response to oxidative stress and metal-induced toxicity [13]. Animal studies have demonstrated that MTs play protective roles in the lungs against multiple oxidative stress-causing factors, such as carmustine [14], ozone [15], paraquat [16], bacterial endotoxins [17], and hypoxia [18]. In contrast, the expression of metallothionein-1 (MT1) is upregulated in different tumors, such as breast cancer, ovarian cancer, urinary bladder cancer, melanoma, and lung cancer [19]. In particular, MT expression level is increased in different types of histological lung cancers, such as squamous cell lung carcinoma and adenocarcinoma [20].

In this study, we analyzed gene expression levels in human pulmonary alveolar epithelial cells (HPAEpiCs) after treatment with PHMG or CMIT. We found that, compared to CMIT, PHMG exposure upregulated the MT1 isomers, including *MT1B*, *MT1F*, *MT1G*, and *MT1H*, in HPAEpiCs. We also identified that metal regulatory transcription factor 1 (MTF1) is an essential transcription factor for the upregulation of MT1B, MT1F, MT1G, and MT1H in HPAEpiCs treated with PHMG, but not in those treated with CMIT.

## 2. Materials and Methods

### 2.1. Chemicals

PHMG (CAS No. 89697-78-9) and CMIT (CAS No. 26172-55-4) were purchased from BOC Sciences (Shirley, NY, USA) and Key Organics-Bionet Research (Camelford, UK), respectively.

### 2.2. Cell Culture

HPAEpiCs were purchased from ScienCell (Carlsbad, CA, USA) and cultured in an alveolar epithelial cell medium (ScienCell) supplemented with 1% penicillin/streptomycin and 10% fetal bovine serum in a 5% carbon dioxide (CO_2_) incubator at 37 °C.

### 2.3. Cell Viability Assay

Cell viability was measured using the Cell Counting Kit-8 (CCK-8; Dojindo, Kumamoto, Japan) in accordance with the manufacturer’s instructions. Briefly, HPAEpiCs (1 × 10^4^ cells) were seeded in a 96-well culture plate (SPL Life Science, Gyeonggi, Korea). After treatment with PHMG and CMIT for 12, 24, and 48 h, the HPAEpiCs were incubated with CCK-8 reagent for 2 h. The absorbance of the wells was measured at 450 nm using a microplate reader (SpectraMax M2e; Bucher Biotec, Basel, Switzerland).

### 2.4. Reverse Transcription-Polymerase Chain Reaction (RT-PCR)

Total RNA was extracted from HPAEpiCs using TRIzol reagent (Invitrogen, Carlsbad, CA, USA). Complementary DNA was synthesized using the amfiRivert cDNA Synthesis Platinum Master Mix (GenDEPOT, Baker, TX, USA) in accordance with the manufacturer’s instructions. Amplification was performed on a thermal cycler (Eppendorf, Hamburg, Germany), and the DNA bands were visualized using a Gel Doc EZ imager (Bio-Rad Laboratories Inc., Hercules, CA, USA). MT1 primers used in this study were designed according to the process described by Mao et al. [21]:

MT1B: sense 5′-GATCCCAACTGCTCCTGCACCACA-3′, antisense 5′-AAGAATGTAGCAAACCGGTCAGGGTAGTT-3′;

MT1E: sense 5′-CACTGGTGTGAGCTCCAGCATCC-3′, antisense 5′-AATGCAGCAAATGGCTCAGTGTTGTATTT-3′;

MT1F: sense 5′-GAATGTAGCAAATGGGTCAAGGTG-3′, antisense 5′-TCTCCTGCACCTGCGCTGGT-3′;

MT1G: sense 5′-CAACTGCTCCTGTGCCGCTGG-3′, antisense 5′-TGTAGCAAAGGGGTCAAGATTGTAGC-3′;

MT1H: sense 5′-ATCTGCAAAGGGGCGTCAG-3′, antisense 5′-GAATGTAGCAAATGAGTCGGAGTT-3′;

MT1M: sense 5′-TCCGGGTGGGCCTAGCAGTCG-3′, antisense 5′-AATGCAGCAAATGGCTCAGTATCGTATTG-3′;

MT1X: sense 5′-GACCCCAACTGCTCCTGCTCG-3′, antisense 5′-GATGTAGCAAACGGGTCAGGGTTGTAC-3′.

### 2.5. Western Blotting

HPAEpiCs were lysed in RIPA lysis buffer (ATTO, Tokyo, Japan). The protein extracts were separated by sodium dodecyl sulfate-polyacrylamide gel electrophoresis and transferred onto a polyvinylidene fluoride membrane (ATTO). After non-specific binding to the membrane was blocked by incubation in 5% skim milk for 1 h, the membrane was incubated overnight with anti-MTF1 (1:1000; Abcam, Cambridge, UK), anti-EGR1 (1:1000; Santa Cruz Biotechnology, Inc., Santa Cruz, CA, USA), anti-nuclear receptor superfamily 3, group C, member 1 (NR3C1; 1:1000; Santa Cruz), anti-Lamin B1 (1:1000; Abcam), and anti-α-tubulin (1:1000; Santa Cruz) antibodies. An appropriate horseradish peroxidase-conjugated secondary antibody (1:2000; Cell Signaling Technology, Danvers, MA, USA) was used to bind to the primary antibodies, and the protein bands were imaged using the ChemiDoc Touch Imaging System (Bio-Rad Laboratories).

### 2.6. Library Preparation and Sequencing

The quality of extracted RNA was assessed using an Agilent 2100 Bioanalyzer and RNA 6000 Nano Chip (Agilent Technologies, Amstelveen, The Netherlands), and RNA quantification was performed using an ND-2000 spectrophotometer (Thermo Fisher Scientific, Inc., Wilmington, DE, USA). For control and test RNAs, library construction was performed using the QuantSeq 3′ mRNA-Seq Library Prep Kit (Lexogen, Inc., Vienna, Austria) in accordance with the manufacturer’s instructions. Briefly, 500 ng of the total RNA was obtained and an oligo-dT primer containing an Illumina-compatible sequence at its 5′-end was hybridized to the RNA to perform reverse transcription. After degradation of the RNA template, second-strand synthesis was initiated using a random primer containing an Illumina-compatible linker sequence at its 5′-end. The double-stranded library was purified using magnetic beads to remove all the reaction components. The library was then amplified to add the complete adapter sequences required for cluster generation. The finished library was purified from other PCR components. High-throughput sequencing was performed as single-end 75 sequencing using NextSeq 500 (Illumina, San Diego, CA, USA).

### 2.7. Data Analyses

QuantSeq 3 mRNA-Seq reads were aligned using Bowtie2 [22]. Briefly, Bowtie2 indices were generated from either the genome assembly sequences or representative transcript sequences to align the reads to the genome and transcriptome, respectively. The alignment file was used to assemble the transcripts, estimate their abundance, and detect the differential expression of genes. The differentially expressed genes were determined based on counts from unique and multiple alignments using BEDtools coverage [23]. The read count data were then processed based on the quantile normalization method using EdgeR within R (Team 2013) and Bioconductor [24]. Genes were classified using the Database for Annotation, Visualization, and Integrated Discovery (DAVID) (http://david.abcc.ncifcrf.gov/ (accessed on 21 November 2018)) and Medline database (http://www.ncbi.nlm.nih.gov/ (accessed on 21 November 2018)) [25].

### 2.8. Statistical Analyses

All data were analyzed using GraphPad Prism v.5.0 (GraphPad Software, San Diego, CA, USA) and are expressed as the mean ± standard deviation. Statistical significance was evaluated using one-way and two-way analyses of variance. Statistical significance was set at *p* < 0.05.

## 3. Results

### 3.1. Measurement of Cell Viability

**Figure 1:**
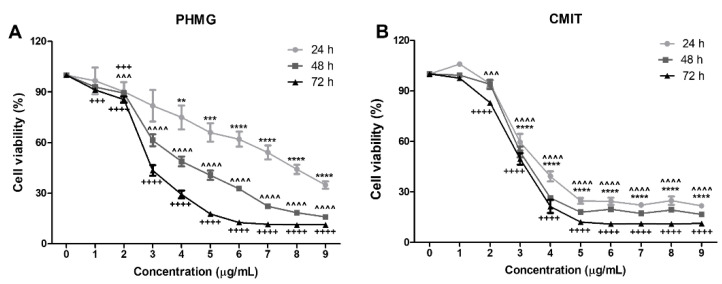
Evaluation of the cell viabilities of human pulmonary alveolar epithelial cells (HPAEpiCs) after exposure to polyhexamethylene guanidine (PHMG) and chloromethylisothiazolinone (CMIT). HPAEpiCs were incubated with various doses of (**A**) PHMG or (**B**) CMIT for 24, 48, and 72 h. Cell viability was measured in triplicate. Statistically significant differences were analyzed using one-way analysis of variance (ANOVA; ** *p* < 0.01, *** *p* < 0.001, **** *p* < 0.0001 versus 0 μg/mL 24 h; ^^^^^ *p* < 0.001, ^^^^^^ *p* < 0.0001 versus 0 μg/mL 48 h; ^+++^ *p* < 0.001, ^++++^ *p* < 0.0001 versus 0 μg/mL 72 h).

To evaluate the cytotoxicity of PHMG and CMIT, HPAEpiCs were treated with different concentrations of PHMG and CMIT for 24, 48, and 72 h, and cell viability was measured. As shown in Figure 1, although there was a slight change in cell viability in HPAEpiCs treated with 2 μg/mL of PHMG and CMIT, HPAEpiCs treated with 3 μg/mL or more showed significantly decreased cell viability. In particular, cell viability was relatively reduced by PHMG exposure in a time-dependent manner, whereas a significant decrease in cell viability was observed after 3 μg/mL CMIT exposure, regardless of the treatment time. Hence, 2 μg/mL of PHMG and CMIT were chosen for further experiments, including gene expression analysis, to investigate the cellular and molecular responses.

### 3.2. Comparative Analysis of Gene Expression after PHMG and CMIT Exposure

**Figure 2:**
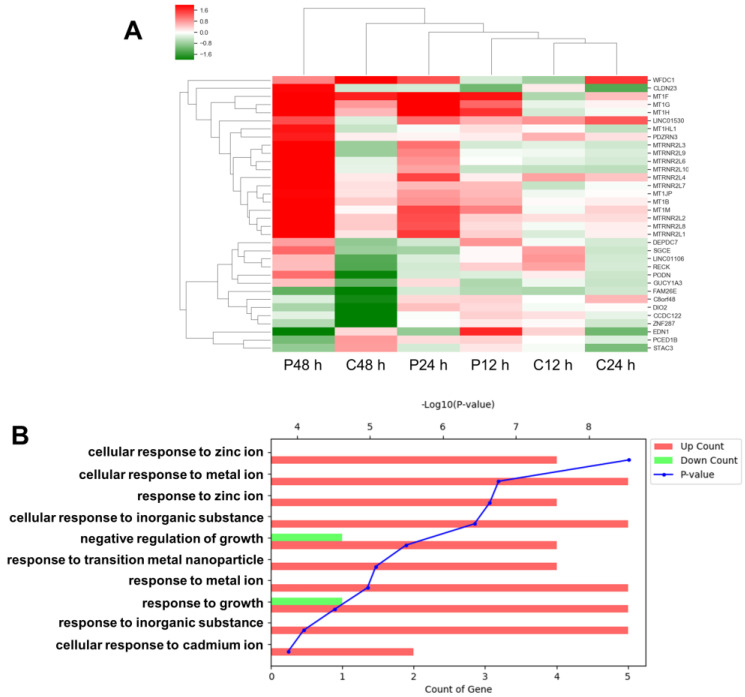
Effects of PHMG and CMIT on the gene expression of HPAEpiCs. (**A**) Heatmap of selected genes (more than 3-fold; *p* < 0.05) in HPAEpiCs after exposure to 2 µg/mL of PHMG or CMIT. (**B**) Gene ontology enrichment analysis of the top 10 biological processes for selected genes.

To date, no comparative study has analyzed the effects of PHMG and CMIT on gene expression. Thus, we performed RNA-Seq to analyze whether gene expression was altered by exposure to PHMG and CMIT. HPAEpiCs were incubated with 2 μg/mL PHMG and CMIT for the indicated time periods prior to gene expression analysis. Overall, the number of genes whose expression changed following exposure to PHMG and CMIT increased markedly over time, by circadian rhythm when exposed for 24 h (Figure S1A,B), and decreased by DNA replication and repair when exposed for 48 h (Figure S1C,D). Interestingly, genes that were significantly upregulated and downregulated after PHMG exposure compared with those after CMIT exposure were selected (more than 3-fold; *p* < 0.05). In total, 34 genes were altered in HPAEpiCs treated with PHMG when compared with those in HPAEpiCs treated with CMIT; of these, a considerable proportion was related to mineral absorption and mitochondrial ribosomal RNA (Figure 2A). Notably, gene ontology enrichment analysis showed that genes involved in cellular responsiveness to Zn^2+^ were significantly upregulated in PHMG-treated HPAEpiCs (Figure 2B). Among the human *MT1* genes [26], our gene analysis revealed that the expression levels of *MT1B*, *MT1E, MT1F*, *MT1G*, *MT1H*, *MT1M,* and *MT1X* were markedly increased in HPAEpiCs treated with PHMG, but not in those treated with CMIT (Figure 2A).

### 3.3. Upregulation of MT1B, MT1F, MT1G and MT1H in Cells Treated with PHMG but Not in Cells Treated with CMIT

**Figure 3:**
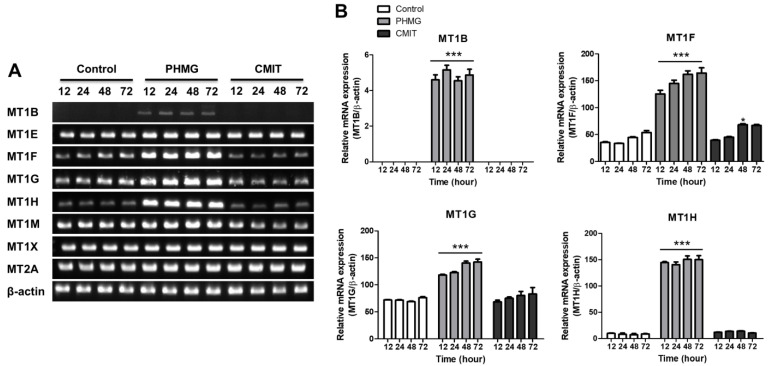
Effects of PHMG and CMIT on the mRNA expression of metallothionein-1 (*MT1*)**.** (**A**) At various time points after exposure to 2 µg/mL of PHMG and CMIT, the mRNA expression levels of *MT1B, MT1E, MT1F, MT1G, MT1H, MT1M, MT1X,* and *MT2A* were analyzed through reverse transcription-polymerase chain reaction (RT-PCR). (**B**) Quantitative analysis of mRNA expression. Statistically significant differences were analyzed by one-way analysis of variance (ANOVA; * *p* < 0.05 and *** *p* < 0.001).

To confirm the mRNA expression of different *MT1* isomers identified in the gene expression analysis, HPAEpiCs were treated with PHMG or CMIT for the indicated time periods (12, 24, 48, and 72 h) and mRNA expression levels of *MT1B, MT1E, MT1F, MT1G, MT1H, MT1M, and MT1X* were measured using RT-PCR. As shown in Figure 3, the mRNA levels of *MT1B, MT1F*, *MT1G*, and *MT1H* were significantly increased in the cells treated with PHMG, but not in those treated with CMIT. No upregulation of *MT1E*, *MT1M*, or *MT1X* was detected in PHMG-treated and CMIT-treated cells. MT2 is a branch of MT1 and is composed of 61 amino acids. MT2 can be distinguished from MT3, which is composed of 68 amino acids and MT4, which is composed of 62 amino acids [27]. The expression of MT2 was also confirmed, but there was no significant difference between the PHMG- and CMIT-treated cells (Figure 3). Collectively, these data indicate that PHMG, but not CMIT, specifically induces intracellular MT1B, MT1F, MT1G, and MT1H.

### 3.4. Role of MTF1 in the Induction of MT1B, MT1F, MT1G, and MT1H

**Figure 4:**
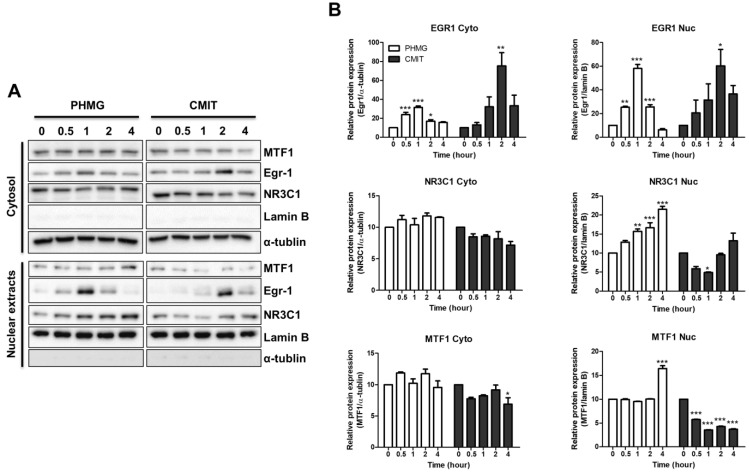
Effects of PHMG and CMIT on the expression levels of MTF1, EGR1, and NR3C1. HPAEpiCs were treated with 2 µg/mL of PHMG or CMIT for the indicated time points. (**A**) Protein levels of metal regulatory transcription factor 1 (MTF1); early growth response 1 (EGR1); nuclear receptor superfamily 3, group C, member 1 (NR3C1); Lamin B; and α-tubulin were measured using Western blotting at 0, 0.5, 1, 2 and 4 h after exposure to PHMG and CMIT. Lamin B and α-tubulin were used as nuclear and cytosol loading controls, respectively. (**B**) Quantitative analysis of the protein levels of MTF1, EGR1, and NR3C1. Statistically significant differences were analyzed by one-way ANOVA (* *p* < 0.05, ** *p* < 0.01, and *** *p* < 0.001).

Previous studies have shown that MTF1 plays a pivotal role in the induction of MTs, which regulate intracellular metal homeostasis [13]. In this study, we observed the nuclear translocation of MTF1 protein by Western blotting after exposure to PHMG and CMIT in HPAEpiCs. In a time-course study, the expression level of translocated MTF1 peaked after 4 h in PHMG-treated HPAEpiCs (Figure 4). In contrast, no nuclear translocation of MTF1 was observed in the CMIT-treated HPAEpiCs, suggesting that the differences in the expression of MT1B, MT1F, MT1G, and MT1H after PHMG and CMIT exposure resulted from differences in transcriptional regulation of MTF1.

The MT1 promoters contain, binding sites for Zn-dependent transcription factors (ZTFs), such as MTF1, EGR1, and NR3C1, which have Zn^2+^-binding motifs [27]. Most MT1 isomers have binding sites for both EGR1 and NR3C1, but MT1F only has an EGR1 binding site and MT1B and MT1H only have NR3C1 binding sites [27]. MT1G also has no binding sites for EGR1 or NR3C1 [27]. Therefore, we hypothesized that the activation of MTF1, which regulates specific MT1 isomer expression, requires the transcriptional activity of EGR1 and NR3C1. To address this hypothesis, we investigated the protein expression levels of EGR1 and NR3C1 after treatment of HPAEpiCs with PHMG and CMIT. Herein, we observed that the nuclear translocation of EGR1 was significantly increased at 1 h and 2 h in HPAEpiCs treated with PHMG and CMIT, respectively. (Figure 4). In addition, the nuclear translocation of NR3C1 was also significantly upregulated after PHMG treatment in a time-dependent manner, whereas the nuclear translocation of NR3C1 was slightly increased at 4 h in CMIT-treated HPAEpiCs (Figure 4). Thus, differences in the expression of MT1B, MT1F, and MT1H after PHMG and CMIT exposure were not significantly affected by EGR1 and NR3C1 transcriptional regulation after PHMG and CMIT exposure, suggesting that EGR1 and NR3C1 may be the basic transcription factors for the upregulation of MT1B, MT1F, and MT1H. Because MT1G has no binding sites for EGR1 or NR3C1 [27], the expression of MT1G could be regulated by MTF1. Taken together, these results indicate that MTF1 may be an essential transcription factor for the induction of MT1B, MT1F, MT1G, and MT1H after exposure to PHMG.

## 4. Discussion

In human and animal studies, the inhalation of PHMG is strongly associated with lung injuries, including interstitial pneumonitis and lung fibrosis, which eventually lead to death owing to the loss of lung function [1,28,29,30]. Lung injuries occur in people exposed to PHMG, a representative HD substance; however, less severe lung damage has been reported in people exposed to CMIT [4]. Nonetheless, the differences in the toxicity mechanisms of chemicals used in HDs and their roles in the development of lung diseases are poorly understood. In this study, we analyzed the differential gene expression of HPAEpiCs after treatment with PHMG and CMIT and elucidated the differences in their molecular responses to these agents.

The results of the analysis of gene expression in human alveolar A549 cells after PHMG exposure have already been reported [10]. However, the previous results are significantly different from our genetic analysis results because of the different cell type and PHMG dose used for genetic analysis. To date, CMIT-induced gene expression has not yet been studied. Therefore, we performed gene expression analysis after treating HPAEpiCs with PHMG and CMIT. Unlike CMIT-induced gene sets, most PHMG-induced gene sets were associated with cellular responses to metal ions, especially Zn^2+^. Zn^2+^ is an essential ion that activates multiple intracellular enzymes and regulates the activity of transcription factors. In particular, methallothionein1 isomers including *MT1B*, *MT1E*, *MT1H*, *MT1HL1*, and *MT1X*, and the membrane transporter gene *SLC30A1*, which controls the concentration of intracellular Zn^2+^ [26], were upregulated in cells exposed to PHMG, while *PARP1*, which is involved in DNA repair [31], was downregulated in HPAEpiCs exposed to PHMG and CMIT (more than 2-fold; *p* < 0.05). Notably, our gene expression analysis data demonstrated that PHMG—but not CMIT—significantly induced the expression of *MT1*, which is capable of binding to metal ions and inhibiting the cellular stress caused by dysregulation of intracellular metal ion concentration [13]. Our results revealed that the mRNA expression of *MT1B*, *MT1F*, *MT1G*, and *MT1H* was markedly upregulated in the HPAEpiCs treated with PHMG but not in those treated with CMIT, suggesting that the suppression of *MT1B*, *MT1F*, *MT1G*, and *MT1H* is responsible for the discrepancy in the cellular responses between the PHMG and CMIT groups. Thus, our results indicate that, compared with CMIT, PHMG significantly increased the expression levels of specific MT1 factors, namely MT1B, MT1F, MT1G, and MT1H.

Several studies have reported that MT1 treatment alleviates lung injury caused by inhaled toxins, such as lipopolysaccharide, Ni, ozone, and paraquat [15,16,17,32]. Obviously, the increase of oxidative stress is detected in the lung tissue of MT-I/II null mice compared to wild-type mice [15,16,18,33], suggesting that MT1 has essential roles in the protection of cells under oxidative stress. Under cellular oxidative stress, MT1 binds with reactive oxygen species and simultaneously releases Zn^2+^ into the cytosol, and the consequent facilitation of the binding of MTF1 and Zn^2+^ [34]. These results suggest that an increase in the expression level of MT1 in cells owing to exposure to PHMG is beneficial for cell survival. In contrast, some lung cancer types, such as large cell lung cancer and non-small cell lung cancer, exhibit MT1 upregulation [19]. In addition, the overexpression of MT1 and MT2 has been detected in tumor tissue samples of lung carcinomas, including squamous cell lung carcinoma and adenocarcinoma [20]. Notably, our recent study showed that bronchiolar-alveolar adenomas are increased in rats after PHMG intratracheal injection [35]. Hence, the upregulation of MT1B, MT1F, MT1G, and MT1H in HPAEpiCs after exposure to PHMG may be involved in the development of lung cancer.

Our results showed that alterations in the expression levels of epithelial mesenchymal transition-related genes associated with cancer progression and metastasis were not significantly different between cells treated with PHMG and CMIT. However, mitochondrial large subunit ribosomal RNA (MTRNR2) was shown to be as much upregulated in cells exposed to PHMG as MT1 (Figure 2). MTRNR2 is a 16S ribosomal RNA in mitochondria that encodes a small open reading frame called humanin (HN), which binds to pro-apoptotic proteins such as Bax, tBid, and BimEL to suppress the induction of cytochrome c, thus inhibiting apoptosis [36,37]. A recent study reported that the additional treatment of HN in mice with breast cancer cells increases spontaneous lung metastasis [38]. In addition, MT1 inhibits apoptosis and promotes cancer cell survival [34]. Therefore, MT1 and MTRNR2 interactions may play an important role in lung cancer development.

There are multiple *cis*-acting elements for ZTFs, such as MTF1, EGR1, NR3C1, specificity protein 1 (SP1), retinoic acid receptor (RAR), Ikaros family zinc finger protein 1 (IKZF1), and churchill domain containing 1 (CHURC1) in the promoter region of MT1 [27]. It is well known that MTF1 plays an important role in the induction of MTs [27]. Among the MT1 isomers, *MT1B* and *MT1H* only have a binding site for NR3C1, whereas MF1F only has a binding site for EGR1. However, MT1G has no binding sites for EGR1 or NR3C1 [27]. Therefore, the possibility of regulating the expression of MT1F via EGR1 and MT1B and MT1H via NR3C1 could not be excluded; thus, the protein levels of EGR1 and NR3C1 were investigated. Our results showed that the expression levels of EGR1 and NR3C1 were increased in the HPAEpiCs treated with PHMG and CMIT, indicating that EGR1 and NR3C1 play a pivotal role in upregulating MT1B, MT1F, and MT1H. In particular, MT1G, which does not have a binding site for EGR1 and NR3C1 [27], could be affected by the transcriptional regulation of MTF1. Thus, our results suggest that the upregulation of MT1B, MT1F, MT1G, and MT1H results from the exposure of cells to PHMG and is regulated by MTF1. However, EGR1 and NR3C1 are not prerequisites for the induction of MT1G upon exposure to PHMG. Although EGR1 and NR3C1 are induced by CMIT exposure, it cannot lead to the upregulation of MTF1, suggesting that MTF1 is a vital transcription factor, along with EGR1 and NR3C1, for the expression of MT1B, MT1F, MT1G, and MT1H.

## 5. Conclusions

Compared to CMIT, PHMG causes two prime cellular responses. First, it induces the synthesis of specific MT1 isomers, including MT1B, MT1F, MT1G, and MT1H. Second, MTF1 is an essential transcription factor that induces the expression of MT1B, MT1F, MT1G, and MT1H. To our knowledge, this is the first study to report the differences in the cellular responses of PHMG and CMIT. The data suggest that the differences in the molecular mechanisms activated by PHMG and CMIT can be used to explain the differences in lung injuries that occur when these agents are inhaled. Therefore, it is necessary to investigate the expression of MT1 isomers and their transcriptional regulation mechanisms after exposure to PHMG and CMIT in future animal studies.

## Data Availability

Data is contained within article.

## Supplementary Materials

The following are available online at https://www.mdpi.com/article/10.3390/toxics9090203/s1, Figure S1: Effects of PHMG and CMIT on the gene expression of HPAEpiCs.
