# Supplementary material for: MTF1 Is Essential for the Expression of MT1B, MT1F, MT1G, and MT1H Induced by PHMG, but Not CMIT, in the Human Pulmonary Alveolar Epithelial Cells
Source: Toxics. 2021 Aug 29;9(9):203. doi: 10.3390/toxics9090203 (PMC8472727; doi:10.3390/toxics9090203)
Supplement: Supplementary file 1 [file toxics-1281924-supplementary.pdf › toxics-1281924-supplementary.pdf]

toxics

MDPI

Supplementary Materials: MTF1 is essential for the expression of MT1B, MT1F, MT1G, and MT1H induced by PHMG, but not CMIT, in the human pulmonary alveolar epithelial cells

Sang-Hoon Jeong, Cherry Kim, Jaeyoung Kim, Yoon-Jeong Nam, Hong Lee, Ariunaa Togloom, Ja-Young Kang, Jin-Young Choi, Hyejin Lee, Myeong-Ok Song, Eun-Kee Park, Yong-Wook Baek, Ju-Han Lee and Ki-Yeol Lee

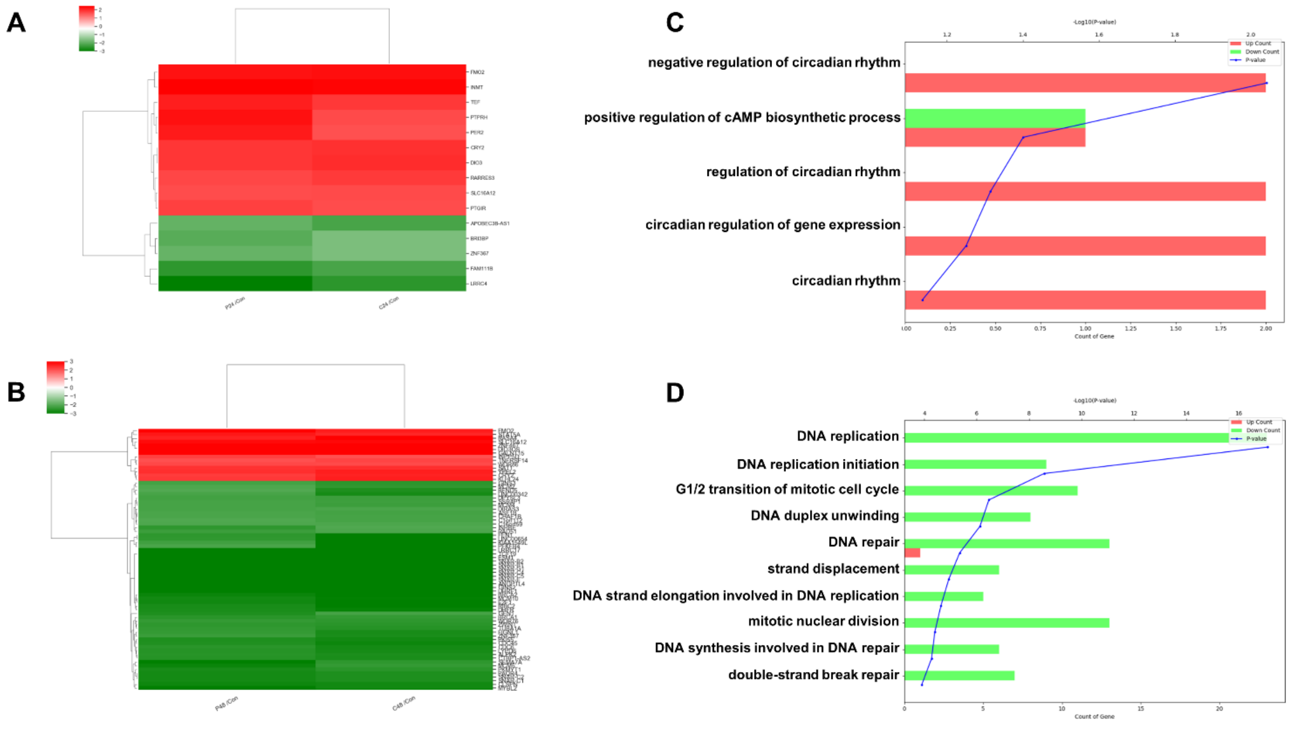

Figure S1. Effects of PHMG and CMIT on the gene expression of HPAEpiCs. (**A**) Heatmap of selected genes (more than 3-fold; $p < 0.05$) in HPAEpiCs after exposure to 2 μg/mL of PHMG or CMIT for 24 h. (**B**) Heatmap of selected genes (more than 4-fold; $p < 0.01$) in HPAEpiCs after exposure to 2 μg/mL of PHMG or CMIT for 48 h. Gene ontology enrichment analysis of the top 10 biological processes for selected genes after exposure to PHMG or CMIT for 24 h (**C**) and 48 h (**D**).
